# Factors associated with problematic internet use among a large sample of Lebanese adolescents

**DOI:** 10.1186/s12887-021-02624-0

**Published:** 2021-03-29

**Authors:** Joseph E. Dib, Chadia Haddad, Hala Sacre, Marwan Akel, Pascale Salameh, Sahar Obeid, Souheil Hallit

**Affiliations:** 1 Division of Psychiatry and Clinical Psychology, School of Medicine, University of Nottingham, Nottingham, UK; 2 Research and Psychology Departments, Psychiatric Hospital of the Cross, P.O. Box 60096, Jal Eddib, Lebanon; 3 INSERM, U1094, Tropical Neuroepidemiology, Limoges, France; 4 Univ. Limoges, UMR 1094, Tropical Neuroepidemiology, Institute of Epidemiology and Tropical Neurology, GEIST, 87000 Limoges, France; 5 CH Esquirol, Department of Psychiatry, 87025 Limoges, France; 6 INSPECT-LB (National Institute of Public Health, Clinical Epidemiology and Toxicology), Beirut, Lebanon; 7 School of Pharmacy, Lebanese International University, Beirut, Lebanon; 8 Faculty of Pharmacy, Lebanese University, Hadat, Lebanon; 9 University of Nicosia Medical School, Nicosia, Cyprus; 10 Faculty of Arts and Sciences, Holy Spirit University of Kaslik (USEK), Jounieh, Lebanon; 11 Faculty of Medicine and Medical Sciences, Holy Spirit University of Kaslik (USEK), Jounieh, Lebanon

**Keywords:** Problematic internet use, Internet addiction, Smoking, Alcohol use disorder

## Abstract

**Background:**

International literature clearly describes factors associated with problematic internet use, including substance dependence, online gambling, social impairment, and functional difficulties. Therefore, it was imperative to assess the extent to which young adolescents in Lebanese schools are affected by problematic internet use (PIU) and the factors associated with it. This large-scale Lebanese survey aims to evaluate the relationship between PIU, depression, and substance use, including alcohol consumption and nicotine use (cigarettes and waterpipe) among adolescents in Lebanese schools.

**Methods:**

This cross-sectional study conducted between January and May 2019 assessed internet use through the Internet Addiction Test (IAT), with ‘severe internet use’ being the threshold for problematic internet use. It enrolled a total of 1810 adolescents aged 14 to 17 from 16 schools from all Lebanese Mohafazat.

**Results:**

The majority of the participants had an average internet use 74.8% (95% Confidence Interval (CI): 0.72–0.76), 20.7% (95% CI: 0.18–0.22) had a frequent internet use, and 4.5% (95% CI: 0.03–0.05) had a severe internet use. Higher alcohol dependence (ß = 0.456, *p* < 0.001), higher depression (ß = 0.079, *p* = 0.001), and having separated parents vs. living together (ß = 0.136, *p* < 0.001) were significantly associated with higher IAT scores. Higher waterpipe dependence (ß = -0.218, *p* < 0.001) was significantly associated with lower IAT scores.

**Conclusion:**

This study, the first and largest of its kind in the Middle East, showed that some psychiatric disorders, such as depression and substance use (smoking and alcohol), are associated with more problematic internet use among Lebanese adolescents. These results could serve as the first step for policymakers towards implementing early awareness campaigns to look at this problem more in-depth and come up with efficient actions to avoid it.

## Background

Internet addiction also referred to as problematic internet use (PIU) in the general literature, is characterized by excessive urges about computer use and internet access, ultimately leading to distress or impairment, notably social and occupational functioning [1]. PIU differs from internet abuse, which refers to improper use of the internet, such as cyber-bullying (the use of the internet to bully and intimidate), and cyber-crime (the use of the internet for unlawful activity, such as hacking and use of computer software for illegal activities). Whether the concept of internet addiction is a disorder is still debated, as the internet allows access to certain addictive activities. For example, gambling is frequent on the internet through online poker and other activities; the argument arises that individuals are not addicted to the internet per se but rather to channels that make the addiction accessible [2].

While the internet has become an essential aspect of our daily life, it is increasingly common among young people and has become a popular tool worldwide [3]. For young adolescents, PIU has been associated with numerous psychological and social problems [4], such as alcohol abuse, smoking, and major depression [5, 6]. As the internet continues to become more pervasive and a staple in daily activities, the prevalence of PIU is expected to rise prominently among young adolescents, prompting more research into this understudied group [7].

The causes of PIU have been advanced into different theoretical frameworks and can be summarized into four main types, which include the Cognitive Behavioral Theory, Social Skills Deficit Theory, Neurobiological Theory, and the I-PACE model [1, 8]. The cognitive-behavioral model proposes that maladaptive cognitions such as self-doubt, self-focused rumination, and low self-efficacy contribute to PIU by promoting behaviors that offer short-term gratification, such as pornography, online shopping, and gambling as seen in individuals with pathological gambling; it also explains the link between depression and internet use [4]. The social skills deficit theory is an explanatory theoretical model stating that individuals with PIU have deficient social skills such as poor social relationships and a negative view of their social competence. Therefore, falling back on computer-mediated interactions grants such individuals greater flexibility in self-presentation compared to face-to-face social interactions. It allows individuals to personify, exaggerate, and fabricate the positive aspects of themselves, thus prompting them to invest long hours and depend solely on online interactions for self-gratification and reinforcing the cycle of compulsive internet use [9]. The neurobiological theories center around disturbed neurotransmitters, specifically serotonin and dopamine. Dopamine has been theorized to play a role in ‘reward dependence’, promoting addictive behaviors, such as gambling and PIU. There is yet to be any research showing direct evidence between dopamine and PIU. However, brain regions associated with addictive behaviors, such as the frontal cortical and subcortical monoaminergic, have shown to be immature in adolescents with PIU, placing them at greater addiction risk [10]. A recent study assessed the relationship between dopamine projection from the ventral tegmental area to the nucleus accumbens and the substantia nigra to the dorsal striatum, which are regions associated with addiction. The results showed a significant relationship between dopamine projection and these brain regions, highlighting that individuals with PIU share similar neuro-biology with other addiction disorders [11]. Finally, the latest and most complex theoretical model associated with PIU is the I-PACE model, which looks at interactions between predisposing factors, moderators, and mediators combined with shortened executive functioning and reduced decision making [8].

The PIU prevalence varies, and several studies reported different prevalence, mainly due to the lack of diagnostic criteria, with the differences in results depending on the measuring tool used. In Lebanon, the PIU prevalence ranged from 16.8% in young school adolescents [12] to 39.1% in university students [13]; these are likely overrated values, given that surveys using screening instruments inherently overestimate prevalence [14].

The relationships of PIU with social (social isolation) and physical variables (headache, backache, dry eyes, neck pain, insomnia) [15] have been extensively studied both in adults and college students. However, psychiatric variables, such as depression and substance use, have received less attention, particularly among primary and secondary level students, where the internet is now highly available.

### Problematic internet use and comorbid disorders

#### PIU and depression

Depression is the leading cause of disability among adolescents worldwide [16]. It is a common mental disorder categorized by profound feelings of sadness, despair, loss of energy, interest, and pleasure, poor appetite, sleep patterns, and concentration, in addition to suicidal ideation and recurrent thoughts of death [17]. There has been substantial work assessing the relationship between PIU and depression [18–21]. While research has shown that it is common among secondary students with PIU, depression prevalence is difficult to estimate, and etiological factors remain inconclusive [22]. Research has pinpointed the cause of depression among school students to several factors such as parental styles, age, genetic predisposition [23], academic performance, lack of exercise [24], and social relationships [25]. Depression in school students increases the risk of developing mental disorders later in life, such as major depression, anxiety, substance use, and suicidal ideation. Early-onset adverse outcomes include poor academic results, education dropouts, and early unplanned pregnancies [26]. A systematic review of 20 studies on PIU and psychopathology showed a strong positive relationship between PIU and depression and other psychiatric comorbid disorders such as anxiety and attention deficit disorder (OR of 1.02 to an OR of 11.66) [27].

#### PIU and substance use

PIU has shown to have a positive relationship with substance use, ranging from legal substances (tobacco, alcohol) to illegal substances (cocaine), in both young and mature adolescents [28, 29]. At its core, it is postulated that since PIU and drug addiction share similar symptoms, they may have a shared underlying neurobiological mechanism responsible for the addictive behavior [30]. As of present, the authors could not locate any studies conducted on Middle Eastern samples examining the relationship between PIU and substance use. Two studies conducted in Turkey and Iran assessed PIU specifically but did not address substance use [31, 32].

#### PIU and alcohol use

Alcohol use is prevalent among young adolescents [33] and associated with PIU [34]; one longitudinal study showed that adolescents with PIU but who did not smoke or drink had heavy drinking and smoking cigarette problems in early adulthood [35].

### PIU and smoking

Research has shown that smoking remains the most prevalent variable associated with PIU, with several studies showing a significant relationship with smoking more than with alcohol in young adolescents [36, 37]. A study among college students in China (*n* = 1092) showed a positive relationship between PIU and substance use, mainly smoking and alcohol use, with higher rates of smoking (10.3%) than alcohol (9.6%). Although conventional smoking (packs, roll-ups, etc.) has been assessed, few studies have explored waterpipe smoking, with one Vietnamese study finding no relationship between waterpipe smoking and PIU [38].

The majority of global research has explored PIU among university students, whether in the USA [39], South Africa [40], South Korea [41], Norway [42], or China [43], with very few focusing on depression and substance use among students aged between 12 and 18. Similarly, studies in Lebanon also examined PIU among university students [13] and explored its relationship with psychiatric disorders, such as depression, but not substance use [12].

Therefore, this study aims to examine the relationship between PIU and depression and substance use in a sample of Lebanese schoolchildren aged 14 to 17, highlighting the associated factors and considering demographic features, such as gender and parental status.

## Methods

### Participants

This cross-sectional study was conducted between January and May 2019, using a proportionate random sample of schools from all Lebanese Mohafazat (Beirut, Mount Lebanon, North, South, and Beqaa) based on the list obtained from the Ministry of Education and Higher Education. A total of 18 private schools was contacted; two refused to participate. Those who accepted were located as follows: 4 in Beirut, 2 in South Lebanon, 6 in Mount Lebanon, 2 in North Lebanon, and 2 in the Beqaa. In each school, all adolescents between 14 and 17 were eligible. Participation was voluntary, and those who enrolled received no financial compensation for their participation. Excluded were those who did not accept to participate. Of the 2000 questionnaires distributed, 1810 (90.5%) were filled and collected back. The methodology used in this study is the same as those used in previous papers [44–47].

### Sample size calculation

The G-power software calculated a minimum sample of 311 participants, based on an effect size f2 = 2%, an alpha error of 5%, a power of 80%, and taking into consideration 8 factors to be entered in the multivariable analysis.

### Procedure

The questionnaire was in Arabic, the native language in Lebanon, and required approximately 60 min to complete. Participants filled out the questionnaire in classrooms to avoid parental influence while answering the questions. Completed questionnaires were handed back to the team and sent for data entry.

### Questionnaire

The first part of the questionnaire assessed the sociodemographic details of the participants (i.e., age, gender, smoking status, parents’ status). The household crowding index was calculated by dividing the number of persons living in the house by the number of rooms, excluding bathrooms and the kitchen [48]. Smoking status was defined in current smokers as smoking daily in the past 30 days.

The second part of the questionnaire included the following scales:

#### Internet addiction test (IAT)

The present study will use the definition of PIU, as stated above. The authors agree that the term internet addiction is limited as it denotes the internet as only negative, whereas PIU semantically describes the internet as a neutral means that can be used problematically, ultimately leading to disruption of the individual psychologically and socially.

The IAT measures the severity of self-reported compulsive use of the Internet in adults and adolescents. The Arabic version validated in Lebanon was used in this study [49]. The IAT consists of 20 items and utilizes a Likert-type scale ranging from 0 (Does not apply) to 5 (Always applies). The final total score varies between 0 and 100, with higher scores indicating higher internet addiction (or PIU in this case). This study applied the same scoring categories used in the original article. The participants were categorized into three levels of internet use: average (scores between 0 and 49), frequent (scores between 50 and 79), and severe (scores between 80 and 100). Those who scored between 80 and 100 (severe) were considered as meeting the criteria for PIU. Cronbach’s alpha was 0.925 in this study [50].

#### The adolescent depression rating scale (ADRS)

The ADRS is a useful, short, clinician-report, and self-report tool developed to screen for depression among adolescents. The ADRS was translated into Arabic using the forward and backward translation method. (One translator translated the scales from English into Arabic, and a second one was involved in the translation from Arabic back into English; discrepancies between the original and translated English versions were resolved by consensus). This 10-item scale is rated by yes/no. Higher scores indicate higher levels of depression [51]. Cronbach’s alpha was 0.940 in this study.

#### The alcohol use disorders identification test (AUDIT)

The AUDIT screening tool created by the World Health Organization (WHO) consists of 10 items and assesses alcohol use, drinking patterns, and alcohol-related problems [52]. This study used the self-report version of the AUDIT validated in Lebanon [47]. The participants were asked to answer the AUDIT part in terms of standard drinks. Scores of 8 or more indicate Hazardous Alcohol Drinking (HAD), while a score < 8 reflects a low risk of alcohol dependence. Cronbach’s alpha was 0.978 in this study.

#### Lebanon waterpipe dependence scale-11 (LWDS-11)

The LWDS-11 constructed and validated in Lebanon is a valid, reliable, and reproducible scale used to assess waterpipe dependence [53]. It consists of 11 items rated a 4-point Likert scale from 0 to 3. Cronbach’s alpha was 0.888 in this study.

#### Fagerström test for nicotine dependence (FTND)

The FTND is a standard instrument for assessing the intensity of physical addiction to nicotine in cigarette smoking. This 6-item tool evaluates the quantity of cigarette consumption, the compulsion to use, and dependence. The validated Arabic version was used in this study [54]. The scoring includes yes/no items scored 0 and 1 and multiple-choice questions scored from 0 to 3. The answers are summed to yield a total score ranging from 0 to 10. The higher the total score, the more intense physical dependence on nicotine [55]. Cronbach’s alpha was 0.825 in this study.

### Statistical analysis

Data were analyzed on SPSS software version 23. The reliability was checked for different factors and the total scales, using Cronbach’s alpha values. Descriptive analyses were done using counts and percentages for categorical variables and mean and standard deviation for continuous measures.

The construct validity of the IAT was done using the principal component analysis. The promax rotation technique was used since the extracted factors were significantly correlated. The Kaiser-Meyer-Olkin measure of sampling adequacy and Bartlett’s test of sphericity were calculated to ensure the model’s adequacy. Factors with eigenvalues greater than one were retained, and the scree plot method was used to determine the number of components to extract. Only items with factor loading greater than 0.4 were considered.

The Shapiro Wilk test was used to check the distribution of normality for the IAT scale and showed that it was normally distributed and not skewed. Thus, the parametric tests were used: the Student t-test to compare between 2 means and the ANOVA for three means or more. In terms of effect size, values of │0.2–0.4│, │0.54–0.7│, and > │0.8│ indicated small, moderate, and large effects. Pearson coefficient was used for linear correlation between continuous variables. For categorical variables, the chi-square and Fisher exact tests were used, as appropriate.

A two-step multiple linear regression analysis was performed, taking the IAT as the dependent variable: model one included only scales, while model two consisted of scales and socio-demographic variables. The absence of multicollinearity was confirmed using the VIF values that were all below 10. All variables that showed a *p* < 0.1 in the bivariate analysis were entered into the model to reduce confounding. Additionally, a multinomial logistic regression was done, taking the categories of internet addiction as the dependent variable to evaluate factors associated with frequent and severe IAT compared to average IAT. In all cases, a value of *P* < 0.05 was considered significant.

## Results

The sociodemographic characteristics of the participants are summarized in Table 1. The mean age was 15.42 ± 1.14 years, 53.3% were females, 74.1% were non-smokers, and 11.9% of the adolescents had separated/divorced parents.

**Table 1:** Sociodemographic characteristics of the sample (*N* = 1810)

	**Frequency (%)**
**Gender**	
Male	844 (46.6%)
Female	963 (53.2%)
Missing data	3 (0.2%)
**Parents status**	
Living together	1581(87.3%)
Separate	213 (11.8%)
Missing data	16 (0.9%)
**Smoking status**	
Yes	395 (21.8%)
No	1411 (78.0%)
Missing data	4 (0.2%)
	**Mean ± SD**
**Age (years) (***N* = 1807)	15.42 ± 1.14
**House crowding index** (*N* = 1799)	1.00 ± 0.64
**Internet addiction test** (*N* = 1789)	39.42 ± 18.08
**Alcohol dependence (AUDIT score)** (*N* = 1724)	6.46 ± 8.44
**Cigarette dependence (FTND score)** (*N* = 1810)	1.52 ± 2.82
**Waterpipe dependence (LWDS-11 score)** (*N* = 1810)	4.72 ± 8.67
**Total depression** (*N* = 1698)	4.64 ± 2.10

**Fig. 1:**
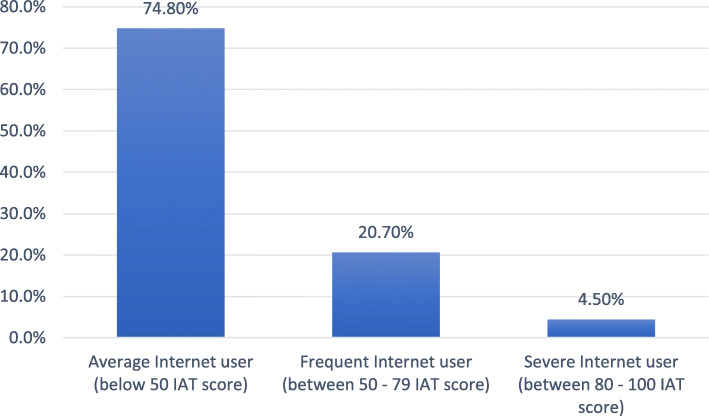
Percentage of internet addiction among participants (*N* = 1810)

The majority of the participants (74.8, 95% Confidence Interval (CI): 0.72–0.76) had an IAT score below 49 (average use), while 20.7% (95% CI: 0.18–0.22) scored between 50 and 79 (frequent use), and 4.5% (95% CI: 0.03–0.05) scored above 80 (severe use) (Fig. 1). PIU was considered for scores in the severe category.

### Factor analysis of the internet addiction test

The factor analysis for the internet addiction test was run over the whole sample. All items could be extracted from the list since no items over-correlated to each other (*r* > 0.9) or had a low loading on factors (< 0.3) or because of a low communality (< 0.3). The IAT items converged over a five-factor that had an Eigenvalue over 1, explaining a total of 69.14% of the variance. A Kaiser-Meyer-Olkin measure of sampling adequacy of 0.891 was found, with a significant Bartlett’s test of sphericity (*p* < 0.001). Table 2 summarizes the components according to the promax rotated matrix. Moreover, Cronbach’s alpha was high for the full test (0.925).

**Table 2:** Factor loading of the internet addiction scale

Items	Question	Factor 1 Preoccupation	Factor 2 Impairment and withdrawal	Factor 3 Tolerance	Factor 4 Social and cognitive aspects	Factor 5 Tolerance and neglect
IAT-12	How often do you fear that life without the internet would be boring, empty, and joyless?	0.837				
IAT-15	How often do you feel preoccupied with the internet when off-line, or fantasize about being online?	0.826				
IAT-13	How often do you snap, yell, or act annoyed if someone bothers you while you are online?	0.735				
IAT-16	How often do you find yourself saying “just a few more minutes” when online?	0.595				
IAT-14	How often do you lose sleep due to being online?	0.559				
IAT-8	How often does your job performance or productivity suffer because of the internet?		0.866			
IAT-6	How often do your grades or school work suffer because of the amount of time you spend online?		0.842			
IAT-5	How often do others in your life complain to you about the amount of time you spend online?		0.560			
IAT-11	How often do you find yourself anticipating when you will go online again?		0.476			
IAT-9	How often do you become defensive or secretive when anyone asks you what you do online?		0.461			
IAT-7	How often do you check your email before something else that you need to do?		0.405			
IAT-17	How often do you try to cut down the amount of time you spend online and fail?			0.801		
IAT-18	How often do you try to hide how long you’ve been online?			0.777		
IAT-19	How often do you choose to spend more time online over going out with others?			0.557		
IAT-20	How often do you feel depressed, moody, or nervous when you are off-line, which goes away once you are back online?			0.526		
IAT-3	How often do you prefer the excitement of the internet to intimacy with your partner?				0.900	
IAT-4	How often do you form new relationships with fellow online users?				0.660	
IAT-10	How often do you block out disturbing thoughts about your life with soothing thoughts of the internet?				0.475	
IAT-1	How often do you find that you stay online longer than you intended?					0.921
IAT-2	How often do you neglect household chores to spend more time online?					0.753
**Cronbach’s alpha**		0.837	0.842	0.838	0.713	0.722
**Percentage of variances explained**		41.69%	9.43%	7.02%	5.89%	5.10%

### Bivariate analysis

The results of the bivariate analysis are summarized in Table 3. Smokers had significantly higher internet addiction compared to non-smokers t (651.09) = − 7.19, *p* < 0.001, d = − 0.404. Higher cigarette (*r* = 0.10, *p* < 0.001), waterpipe (*r* = 0.05, *p* = 0.034), and alcohol dependence (*r* = 0.32, *p* < 0.001) scores were significantly associated with higher IAT score (Table 4).

**Table 3:** Bivariate analysis with problematic internet use as dependent variable

	**Internet addiction test score**	**Effect size d**_**cohen**_	***P*** **-value**
	**Mean ± SD**		
**Gender**			
Male	39.07 ± 18.62	0.036	0.444
Female	39.73 ± 17.59		
**Parents status**			
Living together	39.67 ± 18.44	−0.102	0.130
Separate	37.95 ± 15.04		
**Smoking status**			
Yes	44.95 ± 16.47	−0.404	**< 0.001**
No	37.93 ± 18.21		
	**Correlation coefficient**		***P*** **-value**
Age	**0.052**		**0.027**
House crowding index	−0.012		0.616

**Table 4:** Correlation matrix among the scales used

	Internet addiction test	Alcohol dependence (AUDIT score)	Cigarette dependence (FTND score)	Waterpipe dependence (LWDS-11 score)
Internet addiction test	–			
Alcohol dependence (AUDIT score)	*r* = 0.325, *p* < 0.001			
Cigarette dependence (FTND score)	*r* = 0.108, *p* < 0.001	*r* = 0.576, *p* < 0.001	–	
Waterpipe dependence (LWDS-11 score)	*r* = 0.050, *p* = 0.034	*r* = 0.523, *p* < 0.001	*r* = 0.782, *p* < 0.001	
Total depression	*r* = 0.016, *p* = 0.505	*r* = −0.039, *p* = 0.115	*r* = 0.109, *p* < 0.001	*r* = 0.220, *p* < 0.001

### Multivariable analysis

The results of a first linear regression, taking the IAT score as the dependent variable and the scales used as independent variables, showed that higher alcohol dependence (standardized beta (ß) = 0.441, *p* < 0.001) and higher depression (ß = 0.082, *p* < 0.001) were significantly associated with higher IAT score. Higher waterpipe dependence (ß = -0.205, *p* < 0.001) was significantly associated with lower IAT scores (Model 1, Table 5).

**Table 5:** Multivariable analysis for the IAT variable

	Unstandardized beta	Standardized beta	***p***-value	95% Confidence interval	
				Lower	Upper
**Model 1: Linear regression taking the internet addiction test score as the dependent variable and the scales used as independent variables.**					
Alcohol dependence (AUDIT score)	0.962	0.441	< 0.001	0.838	1.085
Waterpipe dependence (LWDS total score)	−0.420	−0.205	< 0.001	−0.576	− 0.265
Cigarette dependence (FTND total score)	0.152	0.024	0.531	−0.325	0.630
Depression	0.741	0.082	0.001	0.313	1.168
**Model 2: Linear regression taking the internet addiction test score as the dependent variable and the sociodemographic variables and the scales used as independent variables.**					
Alcohol dependence (AUDIT score)	0.994	0.456	< 0.001	0.860	1.128
Waterpipe dependence (LWDS total score)	−0.446	−0.218	< 0.001	−0.603	− 0.290
Cigarette dependence (FTND total score)	0.417	0.066	0.117	−0.104	0.937
Depression	0.714	0.079	0.001	0.286	1.143
Age	0.495	0.031	0.184	−0.235	1.225
Gender (Female vs male^a^)	0.667	0.018	0.437	−1.015	2.349
Parents status (Separate vs living together^a^)	7.485	0.136	< 0.001	4.816	10.153
Smoking status (Yes vs No^a^)	0.351	0.008	0.833	−2.916	3.619

Adjusted R^2^: 0.140

The number of sample included in the first model: 1625

Adjusted R^2^: 0.155

The number of sample included in the second model: 1621

^a^Reference category

The results of a second linear regression, taking the IAT score as the dependent variable and the sociodemographic and the scales used as independent variables, showed that higher alcohol dependence (ß = 0.456, *p* < 0.001), higher depression (ß = 0.079, *p* = 0.001) and having separated parents compared to not (ß = 0.136, *p* < 0.001) were significantly associated with higher IAT score. Higher waterpipe dependence (ß = -0.218, *p* < 0.001) was significantly associated with lower IAT scores (Model 2, Table 5).

The results of the multinomial logistic regression taking frequent vs. average internet use as the dependent variable showed that being a female (Relative Risk Ratio (RRR) = 1.492), being a smoker compared to not (RRR = 3.231), and higher alcohol dependence (RRR = 1.061) were significantly associated with frequent internet use. High cigarette dependence (RRR = 0.875) was significantly associated with lower odds of frequent internet use (Model 1, Table 6).

**Table 6:** Multinomial logistic regression for the level of internet addiction

	*p*-value	RRR	95% CI	
**Model 1: frequent vs average internet use**				
Cigarette dependence (FTND total score)	0.001	0.875	0.805	0.950
Alcohol dependence (AUDIT score)	< 0.001	1.061	1.041	1.082
Smoking status (yes vs no*)	< 0.001	3.231	2.035	5.130
Gender (Female vs Male*)	0.002	1.492	1.158	1.921
Age	0.967	0.998	0.893	1.114
Waterpipe dependence (LWDS-11 total score)	0.173	0.984	0.963	1.007
Parents status (Living together* vs separated)	0.063	0.691	0.468	1.020
**Model 2: severe vs. average internet use**				
Cigarette dependence (FTND total score)	0.037	1.146	1.008	1.304
Alcohol dependence (AUDIT score)	< 0.001	1.099	1.067	1.131
Smoking status (yes vs no*)	0.611	0.814	0.368	1.800
Gender (Female vs Male*)	0.673	0.904	0.566	1.444
Age	0.580	1.063	0.856	1.320
Waterpipe dependence (LWDS total score)	0.002	0.939	0.902	0.976
Parents status (Living together* vs separated)	0.002	1.148	1.044	2.499

**Variables entered in the model:** Age, gender, parents status and smoking status, AUDIT score, LWDS-11 and Fagerstrom

*RRR* Relative Risk Ratio

The number of sample included in the models: 1705

The second model, taking severe vs. average internet use as the dependent variable, showed that having separated parents (RRR = 1.148), higher cigarette dependence (RRR = 1.146), and higher alcohol dependence (RRR = 1.099) were significantly associated with severe internet use. High waterpipe dependence was significantly associated with lower odds of severe internet use (RRR = 0.939) (Model 2, Table 6).

## Discussion

This study aimed to examine the relationship between PIU and depression and substance use in a sample of Lebanese schoolchildren aged 14 to 17. The body of research suggests that PIU is associated with addictive behaviors since PIU symptoms include mood symptoms consistent with withdrawal, greater tolerance with time, and impaired functioning [3, 56].

An in-depth examination of the scales and their psychometric properties identified 45 tools, with the IAT being the most popular and globally validated internet addiction measurement tool, but equally notes a lack of overall factorial consistency [57] justified by cultural differences and methodological issues (i.e., sample size and sociodemographic characteristics) [58]. Previous studies have used the 2, 3, 4, and 5-factor models, with model 2 showing higher consistency than the highest number factor model [57, 59].

Our results revealed that marital status did not significantly affect PIU. Previous findings showed that children growing up in families with separated parents are at a higher risk for developing mental disorders [50, 60, 61]. Although the literature does state substance use and addictive behaviors are more prevalent in children of separated parents [62], it would be worth investigating if PIU, which is a potential gateway to addictive behaviors, manifests differently than other addictive disorders and possibly increases the risk of developing addictive behaviors at a younger age. For example, the legal age to gamble in a casino is between 18 and 21 years of age. However, the internet has less secure gateways where young adolescents can access online gambling sites, potentially exposing them to gambling at a much earlier age.

In our study, higher depression was significantly associated with higher PIU, in line with the literature [19–21]. Several studies have noted that higher levels of depression are associated with higher PIU as individuals become isolated from social interactions in the real world [21, 63, 64]. The age group in this study corresponds to a challenging and stressful phase; indeed, adolescents are burdened with studying (e.g., the stress of exams and time management) and form new relationships, whether romantically or as part of discovering their identity through groups of similar thinking. As these stressful variables possibly increase the risk of depressive states, the internet becomes a gateway to establish risk-free positive communication with other like-minded individuals. Internet escape should not be a replacement therapy for depressed people who feel more secure and in control behind their screens. Those who make a habit of using the internet as a means to escape can end up with depression [63].

Our study examined substance use, specifically alcohol and smoking (cigarettes and waterpipe), and their relation with PIU. The results showed that both alcohol and smoking were significantly associated with PIU, in agreement with the literature, which heavily focuses on older-college level students and not primary/secondary level students as our study [34, 35, 65]. Waterpipe smoking and its relationship to PIU have received little attention in the literature. Our results indicated that higher waterpipe dependence was associated with lower PIU, opposite to the results of a Vietnamese study that showed no association between the two variables [38]. A possible explanation would be that waterpipe smoking is part of the Lebanese culture and that it is considered a leisure activity and a way to socialize with people, giving them less free time for mobile use. Cultural practices are potential indicators, and future studies should consider cultural variants and their relationship with PIU. The general literature has examined substance use, particularly alcohol, and its relationship with PIU, with most studies focusing on young adolescents in the Far East [66]. Substance use usually precedes PIU [65], but the extent to which it does has remained inconclusive due to the sample age, limited to adolescents. However, our results revealed that alcohol use was significantly positively associated with PIU among primary and secondary students [67], in agreement with previous findings showing that harmful alcohol use is associated with PIU. Using the neuro-biological framework, novelty-seeking seen in alcohol use is also present in individuals with PIU. However, more research, specifically in the neurobiological field, is necessary to test the hypothesis that PIU has neurocognitive mechanisms similar to those of substance use disorders.

### Limitations

While the study did take into account a multitude of variables, it has several limitations. The sample may not be representative of the whole Lebanese adolescents as it did not include students from public schools. Some factors related to mood may have been under-reported, such as traits like low self-esteem and sad mood, generally seen as weaknesses in Lebanese culture. The temporal relationship could not be studied because of the cross-section design of the study. Selection bias might have occurred because of the refusal rate (9.5%). Information bias is also possible since students might have over/underestimated the answers to some questions. Residual bias is also likely since some factors (such as socioeconomic status), which might be essential indicators for internet addiction/problematic internet use, were not considered. Moreover, LWDS-11 and ARDS were not validated among Lebanese adolescents. The causes of problematic internet use remain unknown. While theoretical frameworks have been proposed social, cognitive, and neuro-biological standpoints, additional research is necessary to explore these frameworks and their relationship with internet use. For example, do individuals with PIU have the same dopamine-related problems as individuals with gambling addiction?

The IAT used has several flaws; it was developed on an ad-hoc basis, effectively lacking a thorough statistical procedure, and has an unstable factor structure [57]. Additionally, different cut-off scores were proposed but also lacked validation. Lastly, some terms used in the IAT are now outdated. For example, the question ‘Did you check your e-mail before something else you needed to do?’ should be revised, among many other items, as adolescents usually use messenger applications and not e-mail. However, while these shortcomings need to be addressed and rectified accordingly, the IAT remains the most popular scale to assess self-reported PIU. The authors addressed the issue of cut-off scores in this study by only considering those within the ‘severe’ range as having PIU, thus lowering the overestimation of individuals who meet the criteria for internet addiction, as shown in previous Lebanese surveys.

Debates are ongoing regarding the semantics of PIU. Although the term problematic internet use is used concurrently with internet addiction, a consensus has yet to be reached due to uncertainty as to whether internet addiction qualifies as real addiction similar to other substances. An agreement on the terminology would prevent confusion among researchers interested in undergoing research in this field. However, the authors are confident that the definitions of PIU and internet addiction written within this study are concise, clear, and appropriate for future papers addressing PIU.

Nevertheless, our study benefits from a large sample size that extrapolation of the results to the general youth population. Future studies should consider longitudinal designs to investigate whether these associated factors decrease, persist, or intensify into adolescence.

## Conclusion

This study, the first and largest of its kind in the Middle East, showed that some psychiatric disorders, such as depression and substance use (smoking and alcohol), are associated with more problematic internet use among Lebanese adolescents. These results could serve as the first step for policymakers towards implementing of early awareness campaigns to look at this problem more in-depth and come up with efficient actions to avoid it.

## Data Availability

All data generated or analyzed during this study are not publicly available to maintain the privacy of the individuals’ identities. The dataset supporting the conclusions is available upon request to the corresponding author.

## Abbreviations

IAT: Internet addiction test
PIU: Problematic internet use
ADRS: Adolescent depression rating scale
AUDIT: Alcohol use disorders identification test
LWDS-11: Lebanon waterpipe dependence scale-11
FTND: Fagerstrom test for nicotine dependence
